# Canine Myocytes Represent a Good Model for Human Ventricular Cells Regarding Their Electrophysiological Properties

**DOI:** 10.3390/ph14080748

**Published:** 2021-07-29

**Authors:** Péter P. Nánási, Balázs Horváth, Fábián Tar, János Almássy, Norbert Szentandrássy, Norbert Jost, István Baczkó, Tamás Bányász, András Varró

**Affiliations:** 1 Department of Physiology, Faculty of Medicine, University of Debrecen, 4032 Debrecen, Hungary; horvath.balazs@med.unideb.hu (B.H.); tar.fabian@dental.unideb.hu (F.T.); almassy.janos@med.unideb.hu (J.A.); szentandrassy.norbert@med.unideb.hu (N.S.); banyasz.tamas@med.unideb.hu (T.B.); 2 Department of Dental Physiology and Pharmacology, Faculty of Dentistry, University of Debrecen, 4032 Debrecen, Hungary; 3 Faculty of Pharmacy, University of Debrecen, 4032 Debrecen, Hungary; 4 Department of Basic Medical Sciences, Faculty of Dentistry, University of Debrecen, 4032 Debrecen, Hungary; 5 Department of Pharmacology and Pharmacotherapy, Faculty of Medicine, University of Szeged, 6725 Szeged, Hungary; jost.norbert@med.u-szeged.hu (N.J.); baczko.istvan@med.u-szeged.hu (I.B.); varro.andras@med.u-szeged.hu (A.V.); 6 ELKH-SZTE Research Group for Cardiovascular Pharmacology, Eötvös Loránd Research Network, 6725 Szeged, Hungary; 7 Department of Pharmacology and Pharmacotherapy, Interdisciplinary Excellence Centre, University of Szeged, 6725 Szeged, Hungary

**Keywords:** cardiac ion currents, human ventricular cells, canine myocytes, action potential configuration, action potential voltage clamp

## Abstract

Due to the limited availability of healthy human ventricular tissues, the most suitable animal model has to be applied for electrophysiological and pharmacological studies. This can be best identified by studying the properties of ion currents shaping the action potential in the frequently used laboratory animals, such as dogs, rabbits, guinea pigs, or rats, and comparing them to those of human cardiomyocytes. The authors of this article with the experience of three decades of electrophysiological studies, performed in mammalian and human ventricular tissues and isolated cardiomyocytes, summarize their results obtained regarding the major canine and human cardiac ion currents. Accordingly, L-type Ca^2+^ current (I_Ca_), late Na^+^ current (I_Na-late_), rapid and slow components of the delayed rectifier K^+^ current (I_Kr_ and I_Ks_, respectively), inward rectifier K^+^ current (I_K1_), transient outward K^+^ current (I_to1_), and Na^+^/Ca^2+^ exchange current (I_NCX_) were characterized and compared. Importantly, many of these measurements were performed using the action potential voltage clamp technique allowing for visualization of the actual current profiles flowing during the ventricular action potential. Densities and shapes of these ion currents, as well as the action potential configuration, were similar in human and canine ventricular cells, except for the density of I_K1_ and the recovery kinetics of I_to_. I_K1_ displayed a largely four-fold larger density in canine than human myocytes, and I_to_ recovery from inactivation displayed a somewhat different time course in the two species. On the basis of these results, it is concluded that canine ventricular cells represent a reasonably good model for human myocytes for electrophysiological studies, however, it must be borne in mind that due to their stronger I_K1_, the repolarization reserve is more pronounced in canine cells, and moderate differences in the frequency-dependent repolarization patterns can also be anticipated.

## 1. Introduction

In order to better understand the electrophysiology and pathology of the human heart, as well as for the development of new cardioactive agents, reasonably good experimental models of human ventricular cells are needed. In absence of such models, it is difficult to properly interpret the cellular cardiac electrophysiological effects of drugs. Moreover, due to the paucity of proper experimental results, the available in silico human ventricular action potential models [1,2,3,4,5] may still suffer from serious shortcomings. This is basically due to the very limited availability of undiseased human ventricular tissue. Since the early nineties, when the first results on successful isolation of adult human ventricular myocytes were reported [6,7,8], the cells were digested from small tissue chunks excised from the explanted recipient diseased hearts. Accordingly, these studies were performed using the chunk method for cell isolation, but this technique produced a low yield with many injured cells. Human cardiomyocytes can also be derived from pluripotent stem cells [9], but these myocytes carry several features of immature fetal cardiomyocytes, differing markedly from healthy adult ventricular cells in their electrophysiological properties [10,11,12].

An alternative approach is to find the best animal model—possibly among the widely available laboratory animals. However, due to the widespread interspecies differences in the electrophysiological properties of these hearts [13,14,15,16,17,18], a detailed and complex analysis is necessary in this case. Therefore, based dominantly on our earlier experimental results on undiseased human ventricular tissue (obtained by Varró et al. from unused donor hearts after removal of the cuspid valves) using the action potential voltage clamp technique, the profiles of the major human cardiac ion currents (I_Ca_, I_Na-late_, I_Kr_, I_Ks_, I_K1_, I_to1_, I_NCX_) are summarized in the present article. These results are compared to those obtained from similar cells of dogs since the electrophysiological properties of several ventricular ion currents in the two species seem to be similar regarding both their shape and size [17,19,20,21,22,23,24,25]. In addition, the most important interspecies differences observed between ventricular cells of different origins (including human, canine, rabbit, guinea pig, or rat) are also reviewed.

## 2. Significance of the Action Potential Voltage Clamp Technique

The morphology of the cardiac action potential is determined by the finely tuned balance of sequentially activating inward and outward ion currents (for a recent review, see Varró et al. [26]). The amplitude of current at any time depends on the electrochemical gradient (a driving force acting on the ion) and the conductance of the ion channel, governed by its time- and voltage-dependent gating kinetics. Since the membrane potential is continuously changing during the time course of the action potential, the driving force also changes concomitantly. In addition, channel gating has also been shown to be influenced by the dynamics of the membrane potential change (i.e., by the shape of the action potential plateau) [27].

To visualize the actual current profiles conducted during a cardiac action potential, the action potential voltage clamp technique (first applied by Fischmeister et al. in 1984 [28]) was introduced. This technique is essentially based on pharmacological current dissection using the action potential waveform of the cell as a command signal [29]. To date, the action potential voltage clamp method has been successfully applied in a variety of mammalian cardiac cells, including rat [16], porcine [30,31], rabbit [16,30,32,33,34], guinea pig [35,36,37,38,39,40,41], canine [17,23,24,42,43], and human [18,19,20,21,22] myocytes.

## 3. Interspecies Differences in Action Potential Morphology and the Underlying Ion Currents

**Figure 1:**
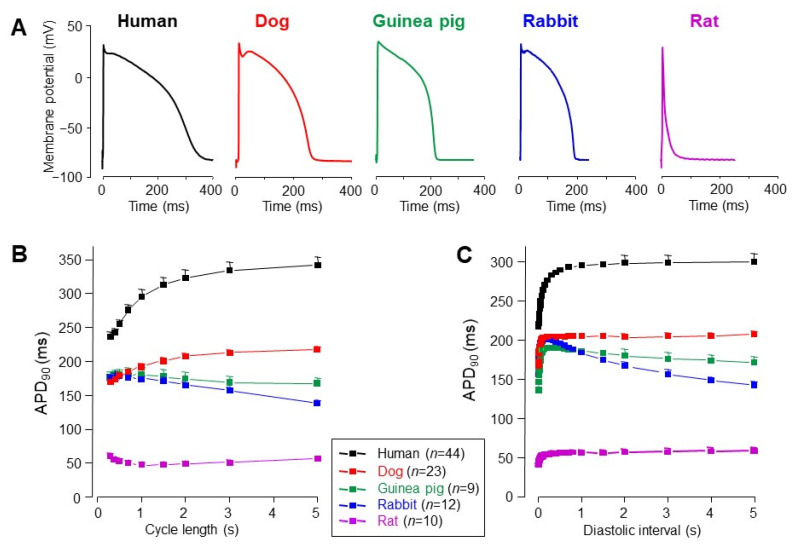
Action potential configurations (**A**), steady-state rate-dependent action potential durations at 90% of repolarization (APD_90_, (**B**)), and APD_90_ restitution relations (**C**) measured in multicellular human, canine, guinea pig, rabbit, and rat ventricular preparations using sharp 3 M KCl-filled microelectrodes. The restitution curves were obtained by gradually increasing the diastolic interval following a train of action potential stimulated at a stable cycle length. Symbols and bars are mean ± SEM values; (*n*) denotes the number of preparations studied [57].

At first glance, there are no striking differences in the morphology of action potentials in recordings taken from multicellular mammalian ventricular preparations—except for small rodents, like mice or rats (Figure 1A). In these latter species, I_to1_, I_Kur_, and I_K1_ are very pronounced, while I_Ca_ inactivates rapidly [44,45,46,47]. As a consequence, there is no room for plateau formation, therefore, action potential duration is extremely short. Furthermore, there is a reverse and slightly biphasic relationship between action potential duration and the pacing cycle length (Figure 1B), in addition to the negative force-frequency relationship, a characteristic of these species. Both are the opposite of those observed in humans, dogs, or guinea pigs, reflecting robust differences in intracellular Ca^2+^ handling. Consequently, electrophysiological results obtained in rat or murine myocytes are often difficult to extrapolate to humans.

In rabbit myocytes, I_to1_ is expressed in epicardial but not in endocardial myocytes [48,49,50]. This I_to1_, mediated dominantly by Kv1.4α channel subunits, is markedly different from canine and human I_to1_, which is largely mediated by the robustly expressed Kv4.3 subunits [51,52,53] with only relatively minor Kv1.4 contribution [52,54]. Importantly, the recovery kinetics of the rabbit type I_to1_ is much slower than the canine and human type, resulting in an inverse or biphasic cycle length–APD relationship, as shown in Figure 1B (see also [55,56,57]). Furthermore, in rabbits, both I_Ca_ and I_Na-late_ display a saddle-like profile under action potential voltage clamp conditions [16,17] in contrast to dogs and humans [17,22,24]. In summary, the ion currents in rabbit ventricular cells and the frequency-dependent behavior of the rabbit ventricular action potential are markedly different from those observed in humans.

Electrophysiological properties of guinea pig ventricular myocytes are also distinctly different from those of canine and human cells. Guinea pig action potentials do not display phase-1 repolarization (Figure 1A). This is because of the lack of I_to1_ in the guinea pig ventricle [13,58] and results in a ramp-like plateau phase. From this point of view, guinea pig cells are different from most mammalian species, including dogs and humans [42,59]. Instead of I_to1_, there is a robust I_Ks_ in the guinea pig ventricle, which is more pronounced than the same current in human or dog [21,60]. Similar to rabbits, I_Ca_ and I_Na-late_ display a saddle-like profile in guinea pigs under action potential voltage clamp conditions [17,18] in sharp contrast to dogs and humans [17,22,24]. Considering these properties, guinea pig myocytes do not seem to be fairly good models of human ventricular cells.

**Figure 2:**
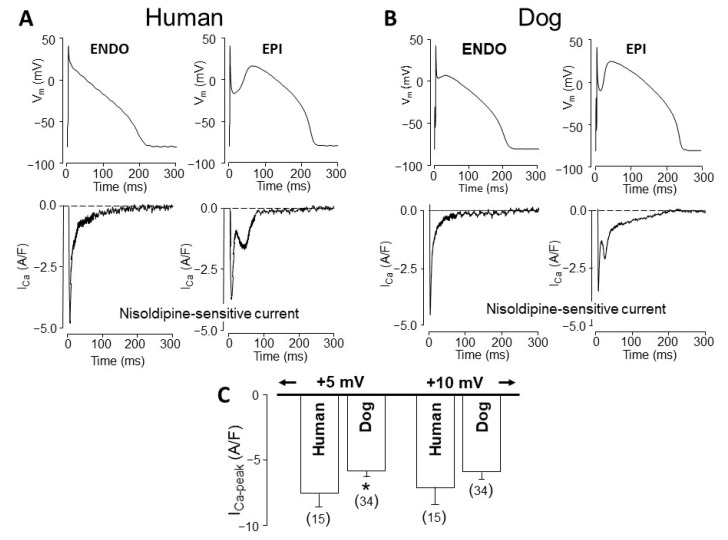
L-type Ca^2+^ currents in human (**A**) and canine (**B**) ventricular myocytes of epicardial (EPI) and endocardial (ENDO) origin. Command action potentials above and I_Ca_ recordings below. I_Ca_ was excised by 1 µM nifedipine. Note the double-peaked I_Ca_ records in EPI and the single-peaked ones in ENDO preparations in both species. (**C**) Comparison of peak current densities between pooled human and canine I_Ca_ obtained under conventional voltage clamp conditions. At test potentials of +5 mV or more negative, significant differences were observed (human I_Ca_ was greater), while no significant differences were found at +10 mV or more positive voltages. Columns and bars are mean ± SEM values, (*n*) denotes the number of myocytes studied, the asterisk (*) indicates significant differences between human and canine I_Ca_ data. (Data from references [21,22,24]).

Pigs are not considered conventionally “laboratory animals”, however, the swine heart is potentially suitable for cardiac transplantation following appropriate immunological modification due to its favorable anatomical properties [61]. Therefore, our analysis would not be complete without discussing the electrophysiological properties of porcine ventricular myocytes. Studying the transmural heterogeneity of action potential morphology, no regional differences were found [62]. Others have identified M cells in the deep regions [63], while others reported transmural inhomogeneity without the identification of M cells [64]. Although the spike-and-dome configuration of the porcine ventricular action potential is similarly shaped to that observed in canine and human myocytes, the pronounced phase-1 repolarization of the porcine action potential is related to the high amplitude of I_to2_, identified as a Ca^2+^-sensitive Cl^-^ current [62]. In contrast to human [65] and canine [66] I_to_, porcine myocytes do not express Kv4.3 channels, and consequently, I_to1_ is absent in these cells [62]. L-type Ca^2+^ current seems relatively weak in porcine myocytes, since the density of 3.6 A/F, measured in porcine myocytes at +10 mV [67], is significantly less than observed by us in humans or canine cells at the same test potential (see Figure 2C). There are only two reports in the literature presenting action potential voltage clamp experiments in swine ventricular cells [30,31]. Comparing these to the respective canine and human data [21,65], it can be concluded that the intensity of I_Ks_ is similar, while densities and integrals of I_K1_ and I_Kr_ are significantly higher in pigs than in dogs or humans. An additional difference between porcine and canine/human myocytes is in the shape of I_Na-late_, since human and canine cells display I_Na-late_ with monotonically decreasing amplitude (“decrescendo” profile), while the shape of porcine I_Na-late_ is saddle-like, showing a “crescendo” profile similar to rabbit and guinea pig I_Na-late_ [17]. These characteristic differences and similarities between human, canine, and porcine myocytes are summarized in the supplement. Although porcine myocytes share several electrophysiological properties with human ventricular cells, the similarity between human and canine cells is superior compared to the same relation between human and porcine myocytes (Table S1, Supplementary Materials).

Although it is not evident from Figure 1, it must be noted that there is a striking difference between the heart rate of all rodents used in the laboratory versus human, canine and porcine hearts. The baseline heart rate in each rodent is much higher than that of larger mammals including humans, pigs, and dogs, which in turn, do not markedly differ from one another in this regard. This is important considering the voltage-, time-, and consequently, rate-dependent gating kinetics of cardiac ion channels.

## 4. Comparison of Human and Canine Ion Currents under Action Potential Voltage Clamp Conditions

**Figure 3:**
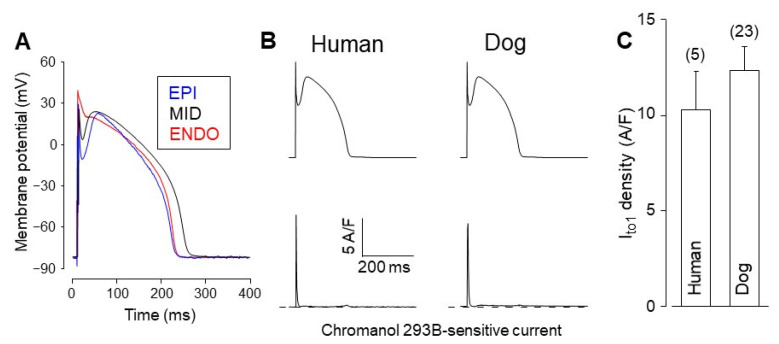
Transient outward K^+^ currents in human and canine left ventricular myocytes. (**A**) Differences in action potential morphology in canine ventricular cells derived from the subepicardial (EPI), midmyocardial (MID), and subendocardial (ENDO) layers. (**B**) Command action potentials (top) and transient outward K^+^ current (I_to1_) records (bottom) taken from midmyocardial human and canine myocytes under action potential voltage clamp conditions. The cycle length of stimulation was 700 ms in the canine and 1000 ms in the human cells. (**C**) Average I_to1_ densities. I_to1_ was defined as a 100 µM chromanol 293B-sensitive current. Columns and bars are mean ± SEM values, (*n*) denotes the number of myocytes studied. Unpublished data from Varró et al.

Due to the considerable interspecies differences discussed above, it seems reasonable to focus on canine myocytes and compare their ion currents to those recorded from undiseased human cells under identical experimental conditions, which is the action potential voltage clamp in the majority of experiments presented in this article. In contrast to the subendocardial myocytes, which are the usually impaled cell type in multicellular studies, the target cell can be freely chosen by its origin or action potential morphology when action potentials are recorded from isolated myocytes. This is demonstrated in Figure 3A by comparing the action potential configuration of canine left ventricular myocytes of subepicardial (EPI), subendocardial (ENDO), and midmyocardial (MID) origin. The asymmetrical transmural distribution of I_to1_, which is manifested in the various magnitudes of phase-1 repolarizations, as shown in Figure 3A, is a common feature of canine and human ventricular myocytes [59,68,69,70,71]. The density of I_to1_ was reported to be 2–5-fold greater in EPI than ENDO cells in canine [70,72] and 3–4-fold greater in human [69,73,74] ventricle depending on the experimental conditions, such as the temperature or test potential. When using identical (EPI-like) command action potentials in midmyocardial human and canine myocytes, no significant differences can be observed in the profiles (Figure 3B) or densities of I_to1_ (Figure 3C). This apparently contradicts previous results obtained under conventional voltage clamp conditions by Akar et al. [52], who pointed out kinetic differences between human and canine I_to1_. Although both human and canine I_to_ is conducted mainly by Kv4.3 channels, the contribution of the Kv1.4 channel was also verified in both canine and human ventricular myocardium [75]. In human ventricular muscle, the majority of the myocytes showed biphasic recovery from inactivation with faster (12.1–13.2 ms) and slower (1197–1283 ms) time constants. These values correspond to the recovery kinetics of Kv4.3 and Kv1.4 channels, respectively [54]. In addition, the Kv channel protein distribution patterns showed transmural heterogeneity reflected also in I_to1_ current densities and action potential configurations [75]. In canine ventricular myocytes, I_to_ also exhibits biphasic recovery from inactivation but with distinctly different faster (28.4–56.6 ms) and slower (177.5–546.6 ms) time constants [76] than those measured in humans. This slight but significant difference in the recovery kinetics is associated with the different degrees of Kv4.3 and Kv1.4 channel protein expression in the two species [77]. In both humans and dogs, the amplitude of I_to_ strongly depends on the level of KChIP2 subunit channel protein expression [77].

It is important to recognize that both in human [54] and dog [78] ventricular muscle, I_to_ “window current” was observed, i.e., there is an overlap between the steady-state activation and inactivation curves in the voltage range between −30 and 0 mV. Therefore, I_to_ can carry small but measurable current during the plateau and early phase 3 repolarization supporting repolarization as part of the repolarization reserve.

Another implication of the EPI–ENDO differences observed in action potential configuration is the differently shaped I_Ca_ profiles in myocytes of EPI and ENDO origin recorded under action potential voltage clamp conditions. As demonstrated in Figure 2, in both species (i.e., in humans and dogs), I_Ca_ displays a double peak profile in EPI, but not in ENDO cells [20,24]. This is the consequence of the different action potential contours since the application of an EPI action potential to an ENDO cell resulted in a double peak-shaped I_Ca_ signal [24]. The density of I_Ca_ was not significantly different in canine and human myocytes when measured at test potentials more positive to +5 mV using conventional voltage clamp protocols, however, the density of I_Ca_ was moderately but significantly greater in humans than in canine cells at membrane potentials of +5 mV or more negative values [21].

**Figure 4:**
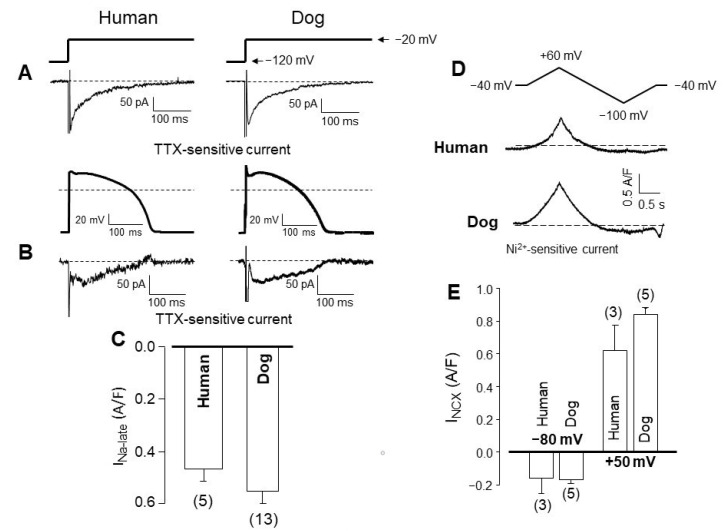
(**A**–**C**) Late Na^+^ current (I_Na-late_) in human and canine ventricular myocytes. I_Na-late_ was recorded under conventional voltage clamp (**A**) and action potential voltage clamp conditions (**B**). The current in this latter case was excised by 10 µM TTX. Pulse protocols are shown above. (**C**) Peak densities of I_Na-late_ measured with action potential voltage clamp in human and canine myocytes. (**D**) Representative I_NCX_, defined as a 10 mM Ni^2+^-sensitive current, recorded using a voltage ramp (−40 mV -> + 60mV -> −100 mV -> −40 mV). (**E**) Peak inward (measured at −80 mV) and outward (measured at +50 mV) NCX current densities. Columns and bars are mean ± SEM values, (*n*) denotes the number of myocytes studied. (Data from references [17,21]).

I_Na-late_, identified in human ventricular cells as a slowly inactivating component of I_Na_ [79], is also very similar in human and canine myocytes compared under either conventional or action potential voltage clamp conditions (Figure 4A,B). I_Na-late_ current densities (determined as currents excised by application of 20 µM tetrodotoxin) are not significantly different in the two species (Figure 4C). More importantly, the decay time constants obtained for I_Na-late_ were also similar: 67 ± 5 and 60 ± 3 ms, respectively, in contrast to the three-fold longer value of 155 ± 16 ms in guinea pig cells [17]. This may explain why I_Na-late_ displays a “decrescendo” profile during the action potential in humans and canines, while a “crescendo” profile in guinea pig myocytes [17].

The current generated by the Na^+^/Ca^2+^ exchanger is difficult to study under action potential voltage clamp conditions. Therefore, it is usually defined as a Ni^2+^-sensitive current measured using voltage ramps of outward or inward directions, representing the reverse and forward mode activities of the exchanger, respectively. The profiles (Figure 4D) and densities (Figure 4E) of I_NCX_ are not significantly different in human and canine myocytes comparing either their inward or outward components [21].

**Figure 5:**
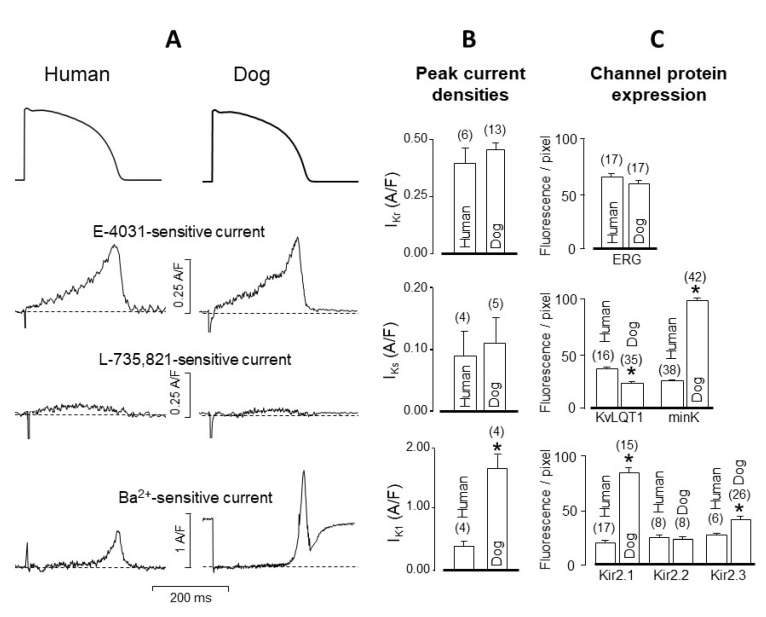
Comparison of peak I_Kr_, I_Ks_, and I_K1_ current densities in human and canine ventricular myocytes under action potential voltage clamp conditions (**A**,**B**). I_Kr_, I_Ks_, and I_K1_ were excised by 5 µM E-4031, 0.1 µM L-735,821, and 500 µM BaCl_2_, respectively. (**C**) Expression of the main channel proteins in human and canine ventricular myocardium. Columns and bars are mean ± SEM values, (*n*) denotes the number of myocytes in (**B**), and myocardial samples in (**C**), the asterisks (*) indicate significant differences between human and canine data. (Data from reference [21]).

As displayed in Figure 5, the shape and density of I_Kr_, defined as an E-4031-sensitive current, as well as the expression of the respective main channel protein, ERG is identical in canine and human ventricular cells. The situation is somewhat different in the case of I_Ks_, defined as an L-735,821-sensitive current since its density was lower in humans than in canine cells when measured using conventional voltage clamp, but no difference was observed in the two species under action potential voltage clamp conditions (Figure 5B, see also [21]). Interpretation of I_Ks_ is further complicated by the asymmetrical expression of the I_Ks_ specific channel proteins since KvLQT1 expression is lower while the expression of minK is higher in canine than in human myocytes (Figure 5C, [21]). Importantly, the amplitude of I_Ks_ is similarly small in both canine and human ventricular myocytes under baseline conditions according to the action potential voltage clamp records shown in Figure 5A,B, therefore its contribution to repolarization is negligible under baseline conditions [80,81]. However, its importance is significant following sympathetic stimulation, when the density of I_Ks_ robustly increases [82,83,84]. Similarly, the relative contribution of I_Ks_ to repolarization increases in both species in case of malfunction of other repolarizing currents (typically I_Kr_) resulting in a longer plateau phase allowing more time for additional I_Ks_ to develop, thus the compensatory contribution of I_Ks_ to the repolarizing reserve can be augmented.

**Figure 6:**
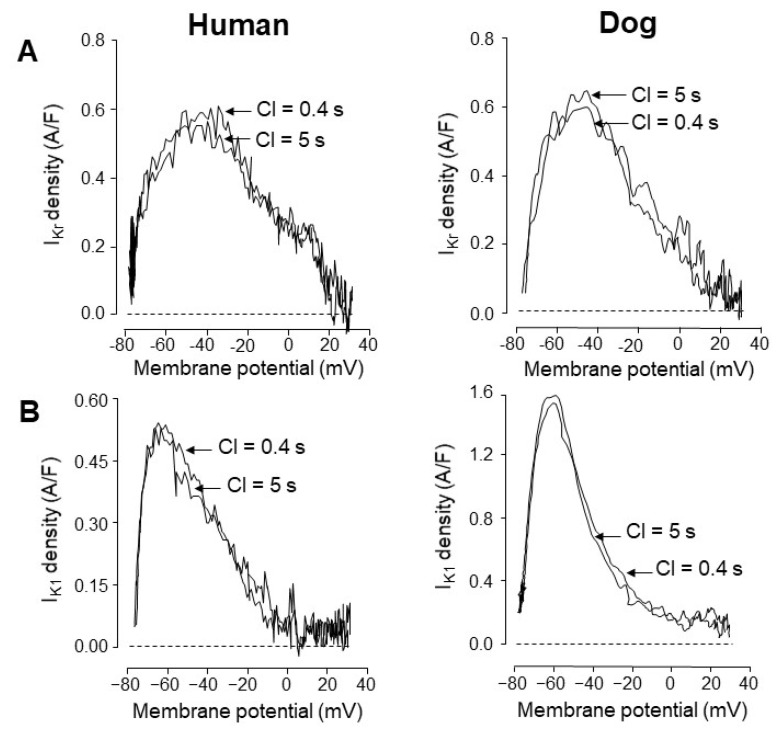
Phase-plane analysis of I_Kr_ (excised by 1 µM E-4031, (**A**)) and I_K1_ (excised by 10 µM BaCl_2_, (**B**)) obtained under action potential voltage clamp conditions in human and canine ventricular myocytes. Representative current–voltage relationships were compared at two different pacing cycle lengths (0.4 and 5 s). The currents showed no rate-dependent properties and displayed similar current–voltage relationships in the two species. Note that the peak amplitude of I_Kr_ was identical in the canine and human myocytes, while the peak amplitude of I_K1_ was three-fold greater in dogs than in humans. (Data from reference [23]).

In contrast to I_Kr_ and I_Ks_, the density of I_K1_ is sharply different in human and canine cells because it is four-fold greater in dogs than in humans (Figure 5A,B and Figure 6B). This is due to the stronger expression of the dominant channel proteins Kir2.1 and Kir2.3 in canine cells (Figure 5C). As a consequence, canine myocytes display a greater repolarization reserve than human cells [21]. This implies that the repolarization lengthening effect of K^+^ channel inhibitor class 3 antiarrhythmic agents is more pronounced in humans than in canine myocytes. This has to be borne in mind when using canine ventricular myocytes to test the repolarization prolonging (side) effect of a new investigational compound for safety pharmacological purposes. As demonstrated in Figure 6, the voltage dependence of the two main K^+^ currents (I_Kr_ and I_K1_) governing terminal repolarization is identical and independent of the pacing cycle length in both species [23]. Accordingly, apart from the higher density of the canine than the human I_K1_, the kinetic properties of the two currents are quite similar in dogs and humans.

**Figure 7:**
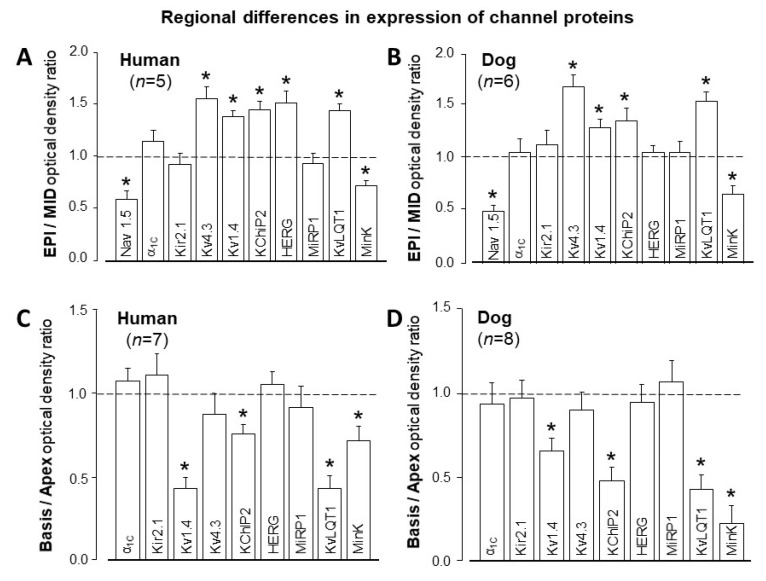
Regional inhomogeneity of the channel protein expression pattern in human and canine ventricular myocardium. (**A**,**B**) Epicardial versus midmyocardial distribution. (**C**,**D**) Apical versus basal distribution. Columns and bars are mean ± SEM values, (*n*) denotes the number of myocardial samples studied, the asterisks (*) indicate significant differences from the ratio of 1. (Data from references [19,20]).

Finally, it is worthwhile to compare human and canine ventricular cells in terms of regional differences in the expression of ion channel proteins. In Figure 7, the two cell types are compared in terms of EPI versus MID and also APEX versus BASIS origin. Differences in the expression patterns of Na^+^ (Nav1.5), Ca^2+^ (α1C), and several K^+^ channel-forming proteins (Kir2.1, Kv4.3, Kv1.4, KChiP2, ERG, MiRP1, KvLQT1, and minK) displayed very similar regional differences in human and canine myocytes [19,20].

## 5. Concluding Remarks

It can be concluded that there is still a severe lack of proper cellular electrophysiological data from undiseased human ventricular tissue. Therefore, further studies are needed in this area to fill this gap, in order to better understand and interpret the translational value of the electrophysiological data obtained in small rodents, guinea pigs, rabbits, and dogs. The available data, however, suggest that—in spite of the modest differences between canine and human ventricular electrophysiology regarding recovery kinetics of I_to1_ and differences in density of I_K1_—it is evident that dog ventricular preparations offer a translational advantage over those of small rodents, guinea pigs, and rabbits. The significant differences in the translational value of data obtained from different animal models should be kept in mind during physiological, pathophysiological, and pharmacological investigations.

## Data Availability

Data sharing not applicable.

## Supplementary Materials

The following are available online at https://www.mdpi.com/article/10.3390/ph14080748/s1, Table S1: Comparison of most important electrophysiological properties of human, canine and porcine ventricular cardiomyocytes.
