# Supplementary material for: Canine Myocytes Represent a Good Model for Human Ventricular Cells Regarding Their Electrophysiological Properties
Source: Pharmaceuticals (Basel). 2021 Jul 29;14(8):748. doi: 10.3390/ph14080748 (PMC8398821; doi:10.3390/ph14080748)
Supplement: Supplementary file 1 [file pharmaceuticals-14-00748-s001.zip › pharmaceuticals-1262239-supplementary.pdf]

pharmaceuticals

MDPI

Supplementary Materials

Table S1. Comparison of most important electrophysiological properties of human, canine and porcine ventricular cardiomyocytes.

Parameter	Human	Canine	Porcine
Transmural inhomogeneity of AP and I_{to}	Yes [1,2,3,8]	Yes [4,5,6,8,9]	No [7]<br>Yes [26,27]
I_{to1}	Kv4.3 dominantKv4.3 [1,3,10,11,12]	Dominant [6,12,13]	Kv4.3 absent [7]
I_{to2}	Absent [11]	Present [13,14,15,16,17]	Present [7]
$I_{Na\text{-}late}$ profile	"decrescendo" [18]	"decrescendo" [18]	"crescendo" [19,20]
I_{Ca}	human = canine > porcine [21,22,23]		
I_{Ks}	human = canine = porcine [19,20,22,24]		
I_{Kr}	human = canine < porcine [19,20,22,24,25]		
I_{K1}	human < canine < porcine [19,20,22,24,25]		

References

1. Näbauer, M.; Beuckelmann, D.J.; Uberfuhr, P.; Steinbeck, G. Regional differences in current density and rate-dependent properties of the transient outward current in subepicardial and subendocardial myocytes of human left ventricle. *Circulation* **1996**, *93*, 168-177.
2. Drouin, E.; Charpentier, F.; Gauthier, C.; Laurent, K.; Le Marec, H. Electrophysiologic characteristics of cells spanning the left ventricular wall of human heart: evidence for the presence of M cells. *J Am Coll Cardiol* **1995**, *26*, 185-192.
3. Wettwer, E.; Amos, G.J.; Posival, H.; Ravens U. Transient outward current in human ventricular myocytes of subepicardial and subendocardial origin. *Circ Res* **1994**, *75*, 473-482.
4. Litovsky, S.H.; Antzelevitch, C. Transient outward current prominent in canine ventricular epicardium but not endocardium. *Circ Res* **1988**, *62*, 116-126.
5. Antzelevitch, C.; Sicouri, S.; Litovsky, S.H.; Lukas, A.; Krishnan, S.C.; Di Diego, J.M.; Gintant, G.A.; Liu, D.W. Heterogeneity within the ventricular wall. Electrophysiology and pharmacology of epicardial, endocardial, and M cells. *Circ Res* **1991**, *69*, 1427-1449.
6. Liu, D.W.; Gintant, G.A.; Antzelevitch, C. Ionic bases for electrophysiological distinctions among epicardial, midmyocardial, and endocardial myocytes from the free wall of the canine left ventricle. *Circ Res* **1993**, *72*, 671-687.
7. Li, G.-R.; Du, X.-L.; Siow Y.L; Karmin, O.; Tse H.-F.; Lau, C.-P. Calcium-activated transient outward chloride current and phase 1 repolarization of swine ventricular action potential. *Cardiovasc Res* **2003**, *58*, 89–98.
8. Szabó, G.; Szentandrássy, N.; Bíró, T.; Tóth, I.B.; Czifra, G.; Magyar, J.; Bányász, T.; Varró, A.; Kovács, L.; Nánási, P.P. Asymmetrical distribution of ion channels in canine and human left ventricular wall: epicardium versus midmyocardium. *Pflügers Arch* **2005**, *450*, 307-316.
9. Li, G.-R.; Lau, C.-P. ; Ducharme, A.; Tardif, J.-C.; Nattel, S. Transmural action potential and ionic current remodeling in ventricles of failing canine hearts. *Am J Physiol Heart Circ Physiol* **2002**, *283*, H1031–H1041.
10. Johnson, E.K.; Springer, S.J.; Wang, W.; Dranoff, E.J.; Zhang, Y.; Kanter, E.M.; Yamada, K.A.; Nerbonne, J.M. Differential expression and remodeling of transient outward potassium currents in human left ventricles. *Circ Arrhythm Electrophysiol* **2018**, *11*, e005914.
11. Li, G.-R.; Feng, J.; Yue, L.; Carrier, M. Transmural heterogeneity of action potentials and I_{to1} in myocytes isolated from the human right ventricle. *Am J Physiol* **1998**, *275*, H369–H377.
12. Akar, F.G.; Wu, R.C.; Deschenes, I.; Armoundas, A.A.; Piacentino, V. 3rd; Houser, S.R.; Tomaselli, G.F. Phenotypic differences in transient outward K^+ current of human and canine ventricular myocytes: insights into molecular composition of ventricular I_{to}. *Am J Physiol Heart Circ Physiol* **2004**, *286*, H602–H609.
13. Zygmunt, A.C.; Robitelle, D.C.; Eddlestone, G.T. I_{to1} dictates behavior of $I_{Cl(Ca)}$ during early repolarization of canine ventricle. *Am J Physiol* **1997**, *273*, H1096–H1106.
14. Zygmunt, A.C. Intracellular calcium activates a chloride current in canine ventricular myocytes. *Am J Physiol* **1994**, *267*, H1984-H1995.

15. Tseng, G.N.; Hoffman, B.F. Two components of transient outward current in canine ventricular myocytes. *Circ Res* **1989**, *64*, 633-647.
16. Váczi, K.; Hegyi, B.; Ruzsnavszky, F.; Kistamás, K.; Horváth, B.; Bányász, T.; Nánási, P.P.; Szentandrássy, N.; Magyar, J. 9-anthracene carboxylic acid is more suitable than DIDS for characterization of calcium-activated chloride current during canine ventricular action potential. *Naunyn Schmiedeberg's Arch Pharmacol* **2015**, *388*, 87-100
17. Horváth, B.; Váczi, K.; Hegyi, B.; Gönczi, M.; Dienes, B.; Kistamás, K.; Bányász, T.; Magyar, J.; Baczkó, I.; Varró, A.; Seprényi, Gy.; Csernoch, L.; Nánási, P.P.; Szentandrássy, N. Sarcolemmal Ca^{2+}-entry through L-type Ca^{2+} channels controls the profile of Ca^{2+}-activated Cl^- current in canine ventricular myocytes. *J Mol Cell Cardiol* **2016**, *97*, 125-139.
18. Horváth, B.; Hézső, T.; Szentandrássy, N.; Kistamás, K.; Árpádffy-Lovas, T.; Varga, R.; Gazdag, P.; Veress, R.; Dienes, Cs.; Baranyai, D.; Almássy, J.; Virág, L.; Nagy, N.; Baczkó, I.; Magyar, J.; Bányász, T.; Varró, A.; Nánási, P.P. Late sodium current in human, canine and guinea pig ventricular myocardium. *J Mol Cell Cardiol* **2020**, *139*, 14-23.
19. Hegyi, B.; Chen-Izu, Y.; Izu, L.T.; Rajamani, S.; Belardinelli, L.; Bers, D.M.; Bányász, T. Balance between rapid delayed rectifier K^+ current and late Na^+ current on ventricular repolarization. An effective antiarrhythmic target? *Circ Arrhythm Electrophysiol* **2020**, *13*, e008130.
20. Hegyi, B.; Bossuyt, J.; Griffiths, L.G.; Shimkunas, R.; Coulibalya, Z.; Jiana, Z.; Grimsrudf, K.N.; Sondergaard, C.S.; Ginsburg, K.S.; Chiamvimonvata, N.; Belardinelli, L.; Varró, A.; Papp, J.G.; Pollesello, P.; Levijoki, J.; Izu, L.T.; Boyd, W.D.; Bányász, T.; Bers, D.M.; Chen-Izu, Y. Complex electrophysiological remodeling in postinfarction ischemic heart failure. *Proc Natl Acad Sci USA* **2018**, *115*, E3036-E3044.
21. Beuckehnann, D.J.; Nabauer, M.; Erdmann, E. Characteristics of calcium-current in isolated human ventricular myocytes from patients with terminal heart failure. *J Mol Cell Cardiol* **1991,** *23*, 929-937.
22. Jost, N.; Virág, L.; Comtois, P.; Ördög, B.; Szűts, V.; Seprényi, Gy.; Bitay, M.; Kohajda, Zs.; Koncz, I.; Nagy, N.; Széll, T.; Magyar, J.; Kovács, M.; Puskás, L.; Lengyel, Cs.; Wettwer. E.; Ravens, U.; Nánási, P.P.; Papp, J.Gy.; Varró, A.; Nattel, S. Ionic mechanisms limiting cardiac repolarization reserve in humans compared to dogs. *J Physiol* **2013**, *591,* 4189–4206.
23. Mukherjee, R.; Hewett, K.W.; Walker, J.D.; Basler, C.G.; Spinale, F.G. Changes in L-type calcium channel abundance and function during the transition to pacing-induced congestive heart failure. *Cardiovasc Res* **1998**, *37*, 432–444.
24. Varró, A.; Baláti, B.; Iost, N.; Takács, J.; Virág, L.; Lathrop, D.A.; Lengyel, C.; Tálosi, L.; Papp, J.G. The role of the delayed rectifier component I_{Ks} in dog ventricular muscle and Purkinje fibre repolarization. *J Physiol* **2000**, *523*, 67-81.
25. Jost, N.; Acsai, K.; Horváth, B.; Bányász, T.; Bitay, M.; Bogáts, G.; Nánási, P.P. Contribution of I_{Kr} and I_{K1} to ventricular repolarization in canine and human myocytes. Is there any influence of action potential duration? *Basic Res Cardiol* **2009**, *104*, 33-41.
26. Stankovicova, T.; Szilard, M.; De, S.I.; Sipido, K.R. M cells and transmural heterogeneity of action potential configuration in myocytes from the left ventricular wall of the pig heart. *Cardiovasc Res* **2000**, *45*, 952–960.
27. Rodriguez-Sinovas, A.; Cinca, J.; Tapias, A.; Armadans, L.; Tresanchez, M.; Soler-Soler, J. Lack of evidence of M-cells in porcine left ventricular myocardium. *Cardiovasc Res* **1997**, *33*, 307–313.
